# Food-grade TiO_2_ is trapped by intestinal mucus in vitro but does not impair mucin *O*-glycosylation and short-chain fatty acid synthesis in vivo: implications for gut barrier protection

**DOI:** 10.1186/s12951-018-0379-5

**Published:** 2018-06-19

**Authors:** Pauline Talbot, Joanna M. Radziwill-Bienkowska, Jasper B. J. Kamphuis, Karine Steenkeste, Sarah Bettini, Véronique Robert, Marie-Louise Noordine, Camille Mayeur, Eric Gaultier, Philippe Langella, Catherine Robbe-Masselot, Eric Houdeau, Muriel Thomas, Muriel Mercier-Bonin

**Affiliations:** 1 Micalis Institute, INRA, AgroParisTech, Université Paris-Saclay, 78350 Jouy-en-Josas, France; 2 Institute of Biochemistry and Biophysics, Polish Academy of Sciences, Pawinskiego 5A, 02-106 Warsaw, Poland; 3 Toxalim (Research Centre in Food Toxicology), Université de Toulouse, INRA, ENVT, INP-Purpan, UPS, Toulouse, France; 4 Institut des Sciences Moléculaires d’Orsay (ISMO), CNRS, Université Paris-Sud, Université Paris-Saclay, Orsay, France; 5 Univ.lille, CNRS, UMR8576-UGSF-Unité de Glycobiologie Structurale et Fonctionnelle, F59000 Lille, France

**Keywords:** Food-grade TiO_2_, Gut barrier, Mucin *O*-glycans, Mucus, Short-chain fatty acids

## Abstract

**Background:**

Titanium dioxide (TiO_2_) particles are commonly used as a food additive (E171 in the EU) for its whitening and opacifying properties. However, the risk of gut barrier disruption is an increasing concern because of the presence of a nano-sized fraction. Food-grade E171 may interact with mucus, a gut barrier protagonist still poorly explored in food nanotoxicology. To test this hypothesis, a comprehensive approach was performed to evaluate in vitro and in vivo interactions between TiO_2_ and intestinal mucus, by comparing food-grade E171 with NM-105 (Aeroxyde P25) OECD reference nanomaterial.

**Results:**

We tested E171-trapping properties of mucus in vitro using HT29-MTX intestinal epithelial cells. Time-lapse confocal laser scanning microscopy was performed without labeling to avoid modification of the particle surface. Near-UV irradiation of E171 TiO_2_ particles at 364 nm resulted in fluorescence emission in the visible range, with a maximum at 510 nm. The penetration of E171 TiO_2_ into the mucoid area of HT29-MTX cells was visualized in situ. One hour after exposure, TiO_2_ particles accumulated inside “patchy” regions 20 µm above the substratum. The structure of mucus produced by HT29-MTX cells was characterized by MUC5AC immunofluorescence staining. The mucus layer was thin and organized into regular “islands” located approximately 20 µm above the substratum. The region-specific trapping of food-grade TiO_2_ particles was attributed to this mucus patchy structure. We compared TiO_2_-mediated effects in vivo in rats after acute or sub-chronic oral daily administration of food-grade E171 and NM-105 at relevant exposure levels for humans. Cecal short-chain fatty acid profiles and gut mucin *O*-glycosylation patterns remained unchanged, irrespective of treatment.

**Conclusions:**

Food-grade TiO_2_ is trapped by intestinal mucus in vitro but does not affect mucin *O*-glycosylation and short-chain fatty acid synthesis in vivo, suggesting the absence of a mucus barrier impairment under “healthy gut” conditions.

**Electronic supplementary material:**

The online version of this article (10.1186/s12951-018-0379-5) contains supplementary material, which is available to authorized users.

## Background

Titanium dioxide (TiO_2_) is widely used as a white pigment and opacifying agent, due to its brightness and high refractive index. It accounts for 70% of the world’s pigment production, with 5000 metric tons produced per year, expected to rise to 60,000 metric tons by 2025. It is used in the food industry in an ultrafine form as a white coloring agent [referred to as food-grade additive E171 in the European Union (EU)] for confectionery, sauces, cakes, and pastries. In the United States, the Food and Drug Administration (FDA) approved the use of food-grade TiO_2_ in 1966 with the stipulation that TiO_2_ should not exceed 1% by weight of the food [1]. In Europe, EU Directive 94/36/EC authorizes the use of E171 in foodstuffs, without establishment of acceptable daily intake by the Joint Food and Agriculture Organization (FAO)/World Health Organization (WHO) Expert Committee on Food Additives, because intestinal TiO_2_ absorption was considered to be very low [2]. A sizable fraction of nano-sized particles (primary particle size < 100 nm) is produced during the manufacturing process of the powder, accounting for 17–55% of the particles present, depending on the commercial supplier of E171 [3–6]. Consumption has been estimated to be 1–2 mg TiO_2_/kg body weight (bw)/day for US children under 10 years of age, and 0.2–0.7 mg TiO_2_/kg bw/day for other US consumers [3]. More recent exposure estimates have ranged between 0.2 and 0.4 mg/kg bw/day in infants and the elderly, and 5.5 and 10.4 mg/kg bw/day in children, depending on the exposure scenario [7].

To date, most data concerning the consequences of TiO_2_ exposure on intestinal barrier function have been obtained in vitro using enterocyte-like cell models [8–15], hence bypassing the mucus layer. However, an increasing body of evidence suggests that mucus plays a key role in gut barrier homeostasis and host protection [16]. Mucus is on the front line, acting in concert with the intestinal epithelium and microbiota, to provide physical, biological, and chemical barriers to potentially harmful food particles [17–20]. However, mucus has only sporadically been taken into account when considering the fate of nanoparticles in the gut, as recently reviewed [21, 22]. In particular, there is only limited information concerning TiO_2_ and oro-gastrointestinal mucus [6, 23–25], even though TiO_2_ nanoparticles were first shown to stimulate mucin secretion by human ChaGo-K1 bronchial epithelial cells in the airways [26]. Porcine mucosa has been used as an ex vivo model for the oral cavity [24, 25], whereas Caco-2/HT29-MTX mucus-producing co-culture has been used to mimic gut-like conditions in vitro [6, 23]. Finally, the interactions between TiO_2_ nanoparticles and mucus have been primarily characterized using standard nano-sized particles (TiO_2_-NP) [23–25], despite their size (primary size and percentage of nano-sized particles) and physico-chemical properties (crystalline structure, surface chemical composition, specific surface area, isoelectric point and agglomeration) which are quite different from those of food-grade TiO_2_ [4, 27]. The sole exception has been the recent study of Dorier et al. [6] on E171.

We performed a mucus-targeted study using food-grade TiO_2_ (E171) in comparison with the NM-105 (Aeroxyde P25) OECD (Organisation for Economic Cooperation and Development) reference nanomaterial. The TiO_2_-trapping properties of mucus were evaluated for E171 using an in vitro approach with mucus-secreting HT29-MTX intestinal epithelial cells. We also compared the TiO_2_-mediated effects in vivo in rats after acute (1-week) or sub-chronic (60 days) oral daily administration of E171 vs. NM-105 at relevant exposure levels for humans (0.1 and 10 mg/kg body weight (bw)/day). We focused on (i) cecal short-chain fatty acids, which were previously shown to be involved in the regulation of intestinal mucin MUC2 expression [28], and (ii) mucin *O*-glycosylation, because it influences the cohesive properties of mucus and hence its protective function [20].

## Methods

### Particle preparation

The E171 sample, obtained from a French commercial supplier of food coloring, was of the same batch as that used in the study of Bettini et al. [5]. NM-105 nanomaterial (also referenced as JRCNM01005a) was provided by the European Union Joint Research Centre (EU JRC) as a test material of manufactured TiO_2_ nanoparticles (Aeroxyde P25) and selected by the OECD for safety evaluation of titanium-based nanomaterials. It displays mixed crystallinity, with anatase as the predominant form (85% anatase:15% rutile), and a primary particle diameter of 22 ± 1 nm [29]. The TiO_2_ products were prepared according to the generic Nanogenotox dispersion protocol described by Jensen et al. [30]. Briefly, a 2.56 mg/mL stock suspension was prepared by pre-wetting the powder in absolute ethanol, followed by dispersion in a 0.05% (w/v) bovine serum albumin (BSA) solution and probe sonication on ice for 27 min at 40% amplitude (Sonifier Cell Disruptor Model 250 20 kHz, Branson Ultrasonics Corporation). The stock suspensions were always prepared fresh prior to each experiment, sonicated, and diluted to the target test concentrations with MilliQ-grade water.

### Particle characterization

The hydrodynamic diameter and polydispersity index of the TiO_2_ particles after the dispersion protocol were measured for E171 and NM-105 at a concentration of 40 µg/mL in Milli-Q grade water by Dynamic Light Scattering (DLS, Zetasizer µV, Malvern Instruments Ltd., UK). Their global electrical surface properties were also assessed by measuring electrophoretic mobility (EM), which corresponds to the velocity of suspended particles under the influence of an applied electrical field. EM was measured in 1 mM KNO_3_ with an automated laser zetameter (Zetaphoremetre II, CAD Instruments, France) under a 100-V electric field. The pH of the suspension was adjusted to between 2 and 7 by adding either nitric acid (HNO_3_, Sigma-Aldrich) or potassium hydroxide (KOH, Sigma-Aldrich). The results are based on an automated video analysis of approximately 200–300 particles for each measurement. The mean velocity of the E171 and NM-105 TiO_2_ particles was determined and expressed as EM in 10^−8^ m^2^/s/V.

### Cell culture

The HT29-MTX mucus-secreting subpopulation of the human colon carcinoma cell line HT29 [31] was kindly provided to the Micalis Institute (MT) by Dr. Thécla Lesuffleur (INSERM UMR S 938, Paris, France). Cells were routinely grown in Dulbecco’s modified Eagle’s minimal essential medium (DMEM) with 4.5 g/L glucose (Lonza, Verviers, Belgium), supplemented with 10% (v/v) fetal calf serum (FCS) (Lonza) inactivated for 1 h at 56 °C, 1% (v/v) l-Glutamine 200 mM (Lonza), and 1% (v/v) penicillin 10,000 U/mL-streptomycin 10,000 µg/mL (Lonza). Cells were seeded at a concentration of 1.2 × 10^5^ cells/mL in µ-Slide 8-well glass bottom slides (Ibidi Biovalley, Nanterre, France) or on glass coverslips placed in 24-well tissue culture plates (Thermo Fisher Scientific—Nunc A/S, Waltham, MA USA) for time-lapse microscopy and fluorescent staining, respectively. Fully differentiated cells were obtained 21 days post-seeding [32]. Cells were maintained at 37 °C in a 10% CO_2_:90% air atmosphere and the culture medium changed daily.

### Fluorescent staining of mucus secreted by HT29-MTX cells

Fluorescent staining was performed as previously described [33] with several modifications. HT29-MTX cells were fixed with 4% paraformaldehyde (PFA) (ThermoFisher Scientific—Fisher Scientific, Waltham, MA USA) for 15 min at room temperature, washed three times with cold phosphate buffered saline (PBS), pH 7.5 (Lonza), and blocked for 90 min at room temperature with 3% (w/v) BSA (Sigma-Aldrich, St. Louis, MO, USA) in PBS. Two primary antibodies (Santa Cruz Biotechnology, Heidelberg, Germany) were used: rabbit anti-MUC5AC (H-160), as MUC5AC is the most prominent gel-forming mucin in this in vitro model [31] and mouse anti-E-cadherin (G-10) to visualize epithelial cells (tight junctions). Both were used overnight at 4 °C at a 1:200 dilution each. Incubation with the secondary antibodies was carried out for 1 h at room temperature using 1:400 diluted anti-rabbit Alexa Fluor 647 and anti-mouse Alexa Fluor 555 goat antibodies (ThermoFisher Scientific—Invitrogen, Waltham, MA USA). After each incubation with antibody, HT29-MTX cells were washed 4 times with cold PBS for 10 min. Stained glass coverslips were mounted on Superfrost microscope slides (ThermoFisher Scientific—ThermoScientific, Waltham, MA USA) using ProLong Gold antifade reagent (Cat. No. P36930, ThermoFisher Scientific—Life Technologies, Waltham, MA USA) and examined on an Leica TCS SP8 AOBS inverted confocal microscope (Leica Microsystems, Mannheim, Germany) equipped with a motorized stage at the INRA MIMA2 platform (http://www.jouy.inra.fr/mima2/). Observations were performed with a 63 ×/1.40 N.A. oil immersion objective. Alexa Fluor 555 was excited at 561 nm using an argon laser and detected between 566 and 628 nm. Alexa Fluor 647 was simultaneously excited at 633 nm using a He/Ne laser and the emitted fluorescence recorded from 638 to 794 nm. Signals were recorded using hybrid detectors (HyD) in standard mode. Single 3D acquisitions were acquired at a scan speed of 600 Hz in bidirectional mode with a scanning zoom of 1, an image resolution of 1024 × 1024 pixels, a line average of 2, and for z-stacks, a z-step between each xy image of 0.3 μm. The images were analyzed, and graphical representations prepared using IMARIS 7.7.2 software (Bitplane, Zurich, Switzerland).

### Confocal laser scanning microscopy of TiO_2_ particles in contact with HT29-MTX cells

#### Visualization of TiO_2_ particle distribution

The distribution of E171 vs. NM-105 TiO_2_ particles at a concentration of 250 µg/mL around HT29-MTX cells was observed using a Leica TCS SP5 confocal laser scanning microscope (Leica Microsystems, France) located at the Centre de Photonique Biomédicale (CPBM) (Orsay, France). The autofluorescence of both cells and particles was excited at 364 nm with a continuous Argon laser and collected between 400 and 700 nm, using a 63× oil immersion objective with a 1.4 numerical aperture. The size of the images was 512 × 512 pixels. Z-stacks were recorded with a z-step of 1 µm from the substratum to the top of the biological structure. To discriminate between TiO_2_ particles and HT29-MTX cells, spectra were reconstructed by imaging the epifluorescence on a photomultiplier with a 10-nm slit, which was moved by 3-nm steps. Spectral deconvolution was performed using the “Dye separation” process of the Leica acquisition software.

#### Visualization of TiO_2_ particle penetration as a function of time

Diffusive penetration of 250 µg/mL TiO_2_ particles through the mucoid structure of HT29-MTX cells was measured by time-lapse microscopy using the same Leica TCS SP5 microscope, as previously described [34]. Briefly, the evolution of fluorescence intensity over time was observed in a focal plane located 18 µm above the glass surface to observe the mucus area. An x–y time series was initiated, in which images were collected every 30 s for 60 min, immediately after TiO_2_ aqueous suspensions were very gently and homogeneously added to the medium above the cells. Images were analyzed using Leica software (Lite; Leica Microsystems, France). Simultaneous transmission imaging showed that no structural alteration of the cells occurred during this process.

### Animals and experimental design

Adult male Wistar rats (175–200 g) were purchased from Janvier Labs (France) and housed in polypropylene cages under standard conditions (temperature 22 ± 2 °C and a 12-h light/dark cycle). Animals were allowed free access to food (standard pellets HARLAN 2018, Envigo RMS SARL, Gannat, France) and water. All experiments were approved by the Local Animal Care and Use Committee (TOXCOM-0036-EH–EH), in compliance with European directive 2010/63/UE. In the first series of experiments, rats were randomly divided into three groups, each containing eight animals, and dosed daily by intragastric gavage (200 μL) for 7 days with food-grade E171, NM-105 (10 mg/kg bw/day), or vehicle (water). In the second series of experiments, rats were randomly divided into three groups, each containing 10 animals, and exposed for 60 days to E171 at the same dose as in the previous experiment or a lower dose (0.1 mg/kg bw/day), through the drinking water, or water alone for the control animals. All rats were decapitated and the cecal contents collected for analysis of short-chain fatty acids and tissues from the small intestine and distal colon were sampled for mucin *O*-glycosylation.

#### Analysis of cecal short-chain fatty acids

The concentrations of short-chain fatty acids (SCFA), namely acetate, propionate, butyrate, valerate, caproate, isobutyrate, isovalerate, and isocaproate, were determined in the cecal contents by gas–liquid chromatography (Nelson 1020, Perkin-Elmer, St Quentin en Yvelines, France) as previously described [35]. SCFA concentrations are expressed in mM.

#### Analysis of gut mucin *O*-glycosylation

Jejunal and/or ileal and distal colonic mucosa were scraped and the mucins solubilized and purified by isopycnic density-gradient centrifugation (Beckman Coulter LE80K ultracentrifuge; 70.1 Ti rotor, 308,500×*g* at 15 °C for 72 h) [36]. The mucin-containing fractions were pooled, dialyzed into water, lyophilized, and further submitted to β-elimination under reductive conditions (0.1 M NaOH, 1 M KBH_4_ for 24 h at 45 °C). Permethylation of the mixture of oligosaccharide alditols was carried out using the sodium hydroxide procedure. After derivation, the reaction products were dissolved in 200 μL methanol and further purified on a C18 Sep-Pak (Waters, Milford, MA). Permethylated oligosaccharides were analyzed by MALDI-TOF Mass Spectrometry (MS) in positive ion reflective mode as [M+Na]^+^. The relative percentage of each oligosaccharide was determined based on the integration of peaks from the MS spectra.

## Results

### Physicochemical characterization of food-grade (E171) vs. model (NM-105) TiO_2_

**Fig. 1:**
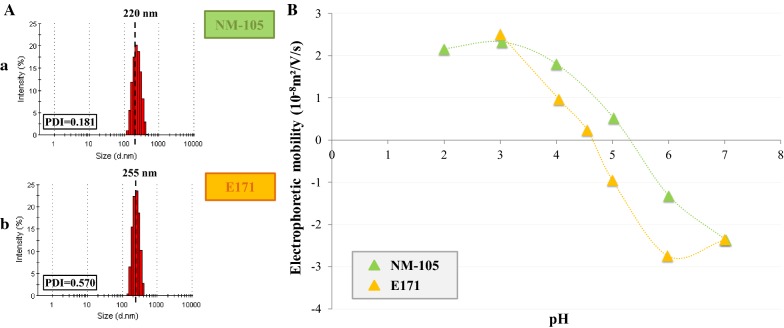
E171 and NM-105 TiO_2_ particle characterization: **A** Hydrodynamic diameter and polydispersity index (PDI) of E171 (a) and NM-105 (b); **B** Electrophoretic mobility of E171 (light orange, triangle) and NM-105 (light green, triangle) at various pH values in 1 mM KNO_3_ supplemented with HNO_3_ or KOH

The distribution of the hydrodynamic diameter after sonication for E171 and NM-105 TiO_2_ particles suspended in Milli-Q grade water was determined by DLS. E171 had a mean hydrodynamic diameter of 255 nm and, under the same conditions, NM-105 exhibited a mean hydrodynamic diameter of 220 nm (Fig. 1A). This indicated that, in both cases, some agglomerates and/or aggregates remained in the suspension, given the primary TiO_2_ particle size measured by transmission electron microscopy, i.e., 22 ± 1 nm (and 100% of particles below 100 nm in diameter) for NM-105 [29] and 118 ± 53 nm (and 44.7% of particles below 100 nm in diameter) for our E171 batch [5]. Figure 1B shows the electrophoretic mobility measurements recorded for E171 and NM-105 in 1 mM KNO_3_ at discrete pH values ranging from 2 to 7. Both types of TiO_2_ exhibited the typical pH-dependence of the electrophoretic mobility of metal oxides, and the isoelectric point (IEP) values for E171 and NM-105 were 4.6 and 5.3, respectively.

### Penetration and localization of TiO_2_ particles within HT29-MTX cells

**Fig. 2:**
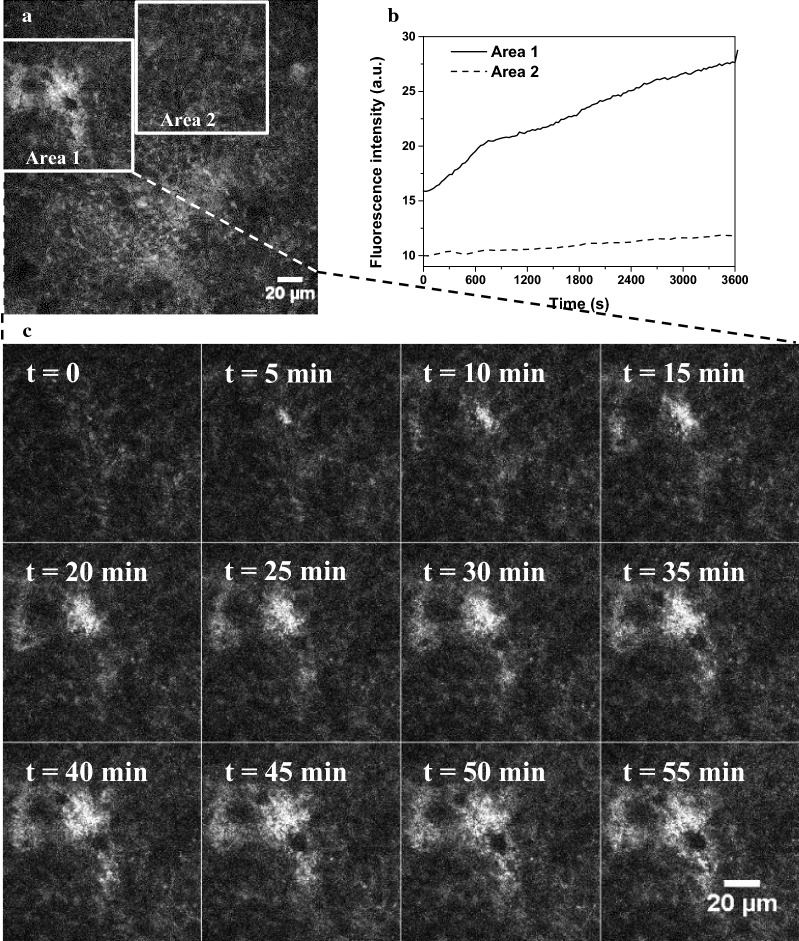
**a** Fluorescence intensity image of HT29-MTX cells in the presence of food-grade E171 particles recorded 18 µm above the substratum 1 h after particle injection. **b** Evolution of fluorescence intensity over time inside two distinct areas identified by white rectangles in the previous image. **c** Fluorescence intensity image sequence over time in area 1 from injection of food-grade E171 to 1 h after. λ_exc_ = 364 nm. λ_em_ = 400–700 nm

**Fig. 3:**
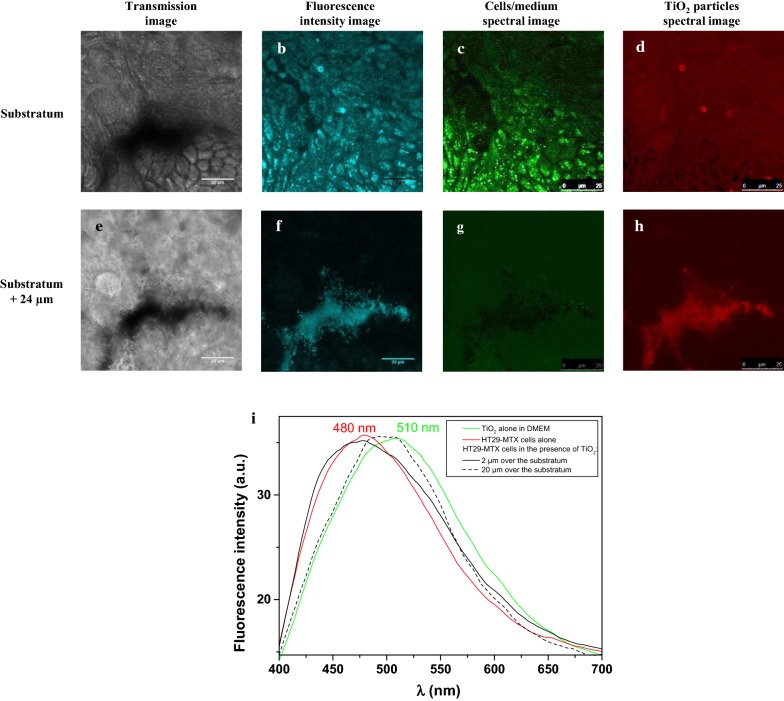
**a** Transmission and **b** fluorescence intensity images of HT29-MTX cells in the presence of food-grade E171 particles close to the substratum. **c** Spectral image of cells/culture medium. **d** Spectral image of E171 particles derived from the deconvolution of the fluorescence intensity image recorded close to the substratum. **e**–**h** Same as for **a**–**d** for a focal plane located 24 µm from the substratum. **i** Normalized fluorescence emission spectra of TiO_2_ particles in cell culture medium (green) and HT29-MTX cells alone in cell culture medium (red) or in the presence of TiO_2_ particles 2 µm (black solid line) or 20 µm above the substratum (black dashed line). λ_exc_ = 364 nm. λ_em_ = 400–700 nm

In contrast to the NM-105 nanomaterial where no fluorescence emission was detected in the conditions under study, food-grade E171, once excited in the near-ultraviolet (UV) range at 364 nm, fluoresced in the visible range with a maximum at 510 nm in the culture medium (Fig. 3i). Based on these intrinsic fluorescence properties, we chose E171 for further time-lapse experiments to visualize the penetration of TiO_2_ particles into the mucoid area of HT29-MTX cells in situ. We observed penetration of food-grade TiO_2_ particles into the mucus and accumulation inside “patchy” regions by setting the focal plane 18 µm above the substratum surface: fluorescence intensity inside some areas increased continuously over time, doubling within 1 h (area 1), whereas it remained almost unchanged in others (area 2) (Fig. 2). Such differences in fluorescence intensity likely indicate region-specific accumulation of TiO_2_ particles. This preferential localization of TiO_2_ particles was confirmed by z-stacks of fluorescence intensity images. Indeed, the only observable signal before E171 addition was cellular autofluorescence in the first micrometers (~ 15 µm) above the substratum (Additional file 1). In contrast, we observed fluorescence deeper, within patches, 1 h after E171 addition (Fig. 2 and Additional file 2). We recorded spectra at different z-positions to further characterize this fluorescence pattern (Fig. 3). Near the substratum, the spectrum displayed a maximum at 480 nm, similar to that for the cells alone, whereas 20 µm above the substratum (i.e., in the region of high fluorescence intensity), the spectrum was identical to that obtained with food-grade TiO_2_ alone in the cell culture medium (Fig. 3i). The spectral images recorded in these different focal planes were then deconvoluted to separate the fluorescence of TiO_2_ from that of the cells (Fig. 3b–d, f–h). The fluorescence near the substratum mainly originated from the cells, whereas in the upper layers it originated from the TiO_2_ particles, which accumulated into “islands”, while the surrounding medium contributed very little to global fluorescence (see the black background in Fig. 3f). TiO_2_ accumulation was also visualized in the transmission images (Fig. 3a, e) as black shadows, because light cannot pass through TiO_2_ particles.

**Fig. 4:**
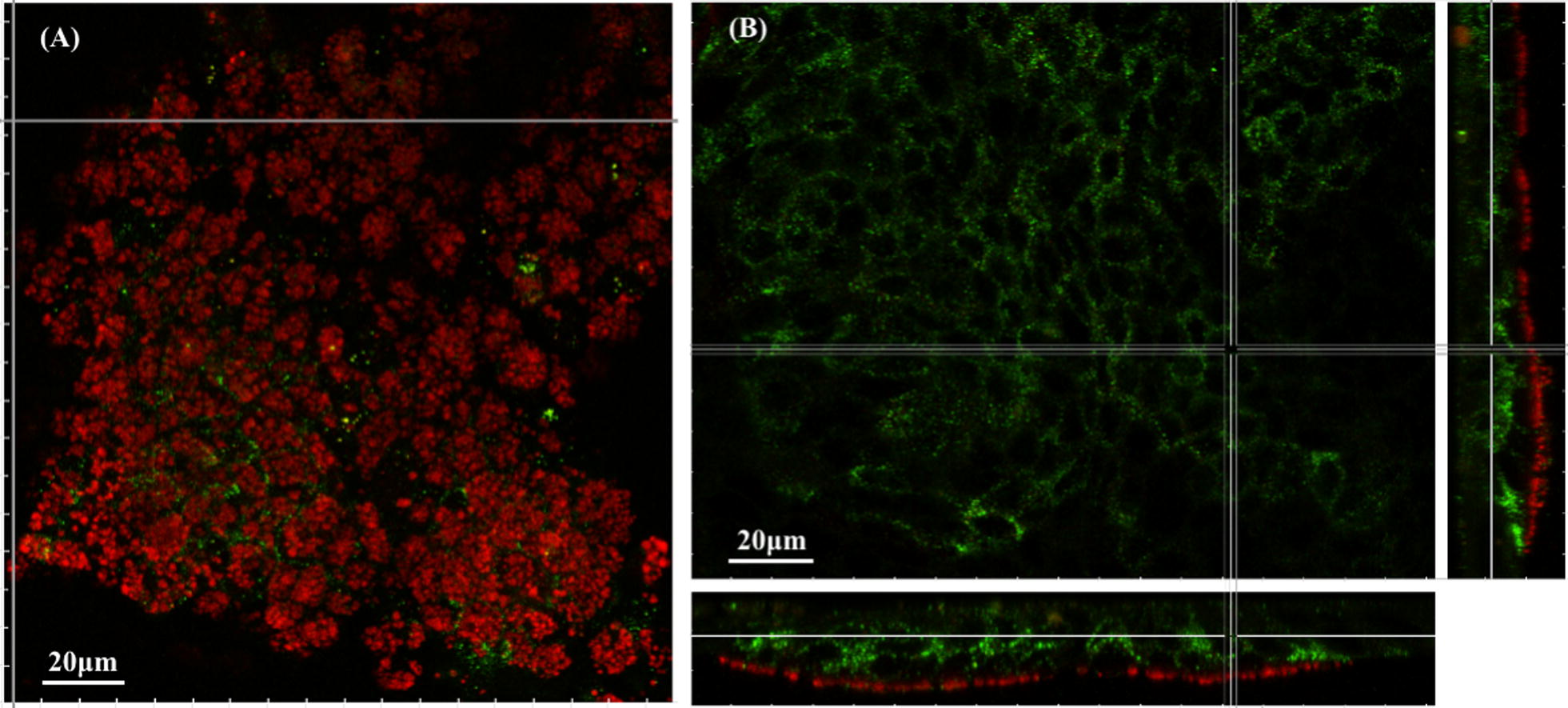
MUC5AC immunofluorescence staining of the HT29-MTX cell line. Representative confocal laser scanning microscopy z-stack image of MUC5AC (in red) produced by HT29-MTX cells (E-cadherin, in green) cultured under standard conditions. **A** z-stack section (x, y) at the level of the mucus; **B** z-stack section (x, y) at the level of HT29-MTX cells with orthogonal x (y, z) and y (x, z) sections on the right and bottom, respectively. The image was prepared using the easy 3D section view of IMARIS software with extended view (signal gathered from a thickness of 5 µm)

We investigated the region-specific accumulation of E171 TiO_2_ particles by characterizing the structure of the mucus produced by HT29-MTX cells by MUC5AC immunofluorescence staining followed by confocal laser scanning microscopy (Fig. 4). We observed a thin layer of mucus, with a thickness of less than 5 µm (Fig. 4B). Furthermore, this mucus layer did not homogenously cover the cells underneath, but was rather organized as regular “islands”, located approximately 20 µm above the substratum (Fig. 4A, B), consistent with the TiO_2_ accumulation pattern depicted above. Thus, the region-specific trapping of food-grade TiO_2_ may be related to the patchy structure of the mucus, even though these fluorescence experiments do not provide direct experimental evidence.

### TiO_2_ oral exposure in rats: impact on cecal short-chain fatty acids and mucin *O*-glycosylation

We next evaluated TiO_2_-mediated effects in vivo after oral exposure, depending on TiO_2_ type (E171 vs. NM-105), treatment duration (1-week vs. sub-chronic 60 days), and the exposure level (0.1 mg/kg bw/day vs. 10 mg/kg bw/day). We focused on the synthesis of cecal short-chain fatty acids (SCFA) and gut mucin *O*-glycosylation.

**Fig. 5:**
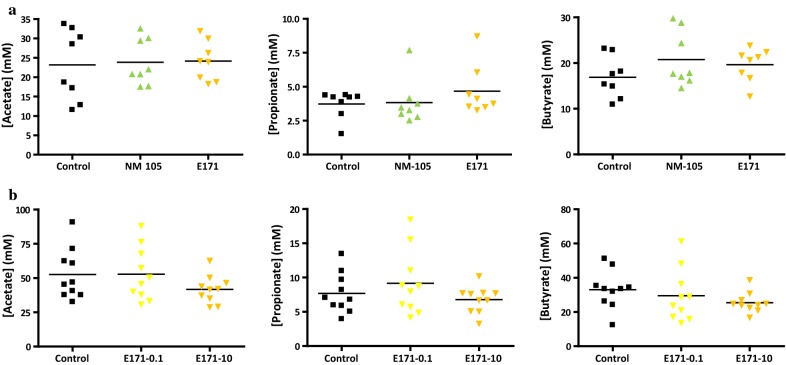
Effect of TiO_2_ exposure in rats on the cecal composition of acetate, propionate, and butyrate after (**a**) a 7-day treatment (black: control; light green: NM-105 10 mg/kg bw/day; light orange: E171 10 mg/kg bw/day) and (**b**) a 60-day treatment (black: control; yellow: E171 0.1 mg/kg bw/day; light orange: E171 10 mg/kg bw/day). Points are from individual rats and bars represent the mean. Statistical significance, assessed using GraphPad Prism 4 software, was determined by one-way ANOVA followed by post hoc tests. Significance was set at a *p* value < 0.05

**Fig. 6:**
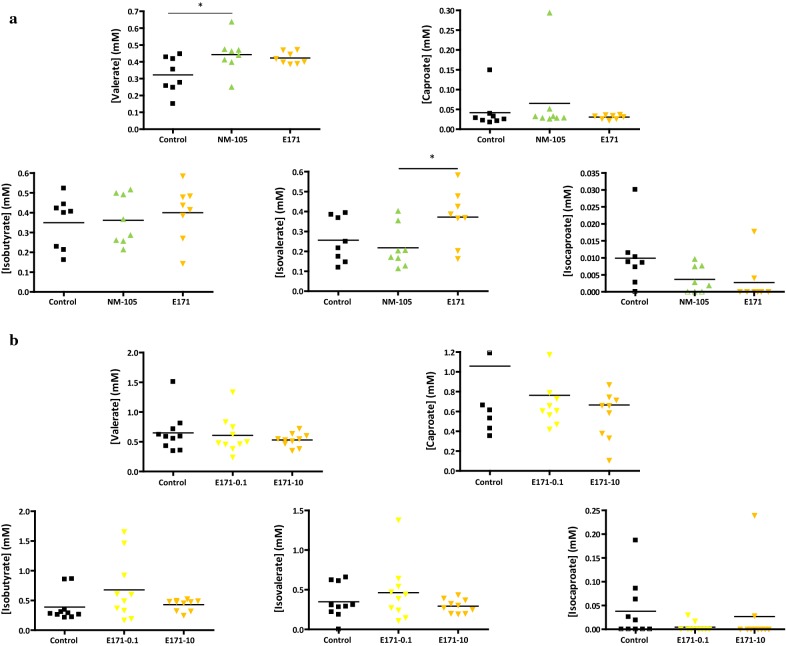
Effect of TiO_2_ exposure in rats on the cecal composition of valerate, caproate, isobutyrate, isovalerate, and isocaproate after (**a**) a 7-day treatment (black: control; light green: NM-105 10 mg/kg bw/day; light orange: E171 10 mg/kg bw/day) and (**b**) a 60-day treatment (black: control; yellow: E171 0.1 mg/kg bw/day; light orange: E171 10 mg/kg bw/day). Points are from individual rats and bars represent the mean. Statistical significance, assessed using GraphPad Prism 4 software, was determined by one-way ANOVA followed by post hoc tests. Significance was set at a p value < 0.05

**Fig. 7:**
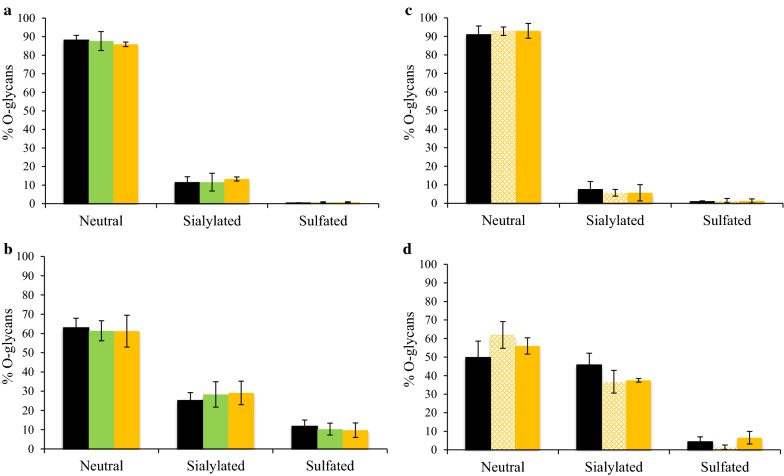
Effect of TiO_2_ exposure in rats on the relative percent of neutral, sialylated, and sulfated mucin *O*-glycans after (**a**, **b**) a 7-day treatment (black: control; light green: NM-105 10 mg/kg bw/day; light orange: E171 10 mg/kg bw/day) and (**c**, **d**) a 60-day treatment (black: control; light orange hatched: E171 0.1 mg/kg bw/day; light orange: E171 10 mg/kg bw/day) in (**a**, **c**) small intestine (**a**: ileum; **c**: jejunum) and (**b**, **d**) distal colon. The results shown are average values and standard deviations for three pools of three to four samples for each condition

A 7-day oral exposure of rats to TiO_2_ had little to no impact on overall cecal composition of SCFA relative to controls, irrespective of TiO_2_ type, E171 or NM-105 (Figs. 5a and 6a). There were also no significant differences relative to controls for acetate, propionate, butyrate (Fig. 5b), or other SCFAs (Fig. 6b) following sub-chronic oral exposure (60 days) with food-grade TiO_2_ in the drinking water at doses of either 0.1 or 10 mg/kg bw/day, i.e., doses approximating human dietary levels for adults and children [3, 7]. In addition, there was no effect relative to controls on mucin *O*-glycosylation in the small intestine of the rats following 7- or 60-day TiO_2_ oral exposure, regardless of TiO_2_ type (E171 and NM-105) or E171 dose tested (Fig. 7a, c). Indeed, there were no substantial modifications for neutral (the most abundant structures), sialylated, or sulfated oligosaccharides. The proportion of neutral *O*-glycans was lower in the colon of control animals than in the small intestine, which was associated with a higher amount of sialylated and sulfated structures (Fig. 7). There were also no TiO_2_-induced changes in colonic *O*-glycans after either a 1-week or sub-chronic treatment relative to control conditions (Fig. 7b, d).

## Discussion

The whitening and opacifying properties of TiO_2_ are commonly exploited when it is used as the food additive E171. However, the possibility of gut barrier disruption and/or an intestinal homeostatic imbalance (including microbiota dysbiosis) has been of increasing concern, due to the presence of a nano-sized fraction in this additive [4, 27]. Indeed, recent studies have reported adverse effects of in vitro exposure of intestinal epithelial cells to E171 [6, 11, 37] some of which possibly predispose the host to intestinal diseases and colorectal cancer, as shown in rodents [5, 38]. Intestinal mucus is still an underestimated gut target in food nanotoxicology [21, 22] and little data is currently available concerning the effects of TiO_2_. Most available data have been generated using in vitro cell models, exclusively studying the effects of TiO_2_ nanoparticles (TiO_2_-NP) [23, 26]. Various profiles for the absorption and transport of TiO_2_-NP have been described, depending on whether epithelial cells are cultured alone or with goblet cells [23]. Intracellular TiO_2_-NP accumulation has been mapped through Ti-distribution imaging by particle-induced X-ray emission. The authors showed that Caco-2 cells alone in monoculture displayed low levels of intracellular Ti accumulation after 24 h of contact. In contrast, the same treatment in the presence of goblet cells (Caco-2/HT29-MTX mucus-producing co-culture) led to 50-fold higher levels of intracellular accumulation [23], suggesting facilitated translocation of TiO_2_-NP in the presence of mucus. In contrast, another study using the same type of TiO_2_-NP, but Inductively Coupled Plasma Mass Spectrometry (ICP-MS) to measure Ti content, showed that Ti accumulation in Caco-2 cells after 6 or 48 h of contact was slightly higher than in Caco-2/HT29-MTX cells; however, the differences were not statistically significant. The same results were obtained after repeated exposure for 21 days [6]. Ex vivo studies on porcine buccal mucosa also reported that TiO_2_-NP were able to permeate the mucus layer and penetrate the underlying tissue, regardless of their size and hydrophilicity/hydrophobicity [24, 25].

Physico-chemical analysis of E171 revealed a hydrodynamic diameter of 255 nm, which was of the same order of magnitude as values obtained for the same commercial source [5] and other food-grade TiO_2_ samples [4], where effects of sonication protocols may influence the size of agglomerates [39]. E171 was also found to exhibit the typical pH-dependence of electrophoretic mobility of metal oxides, in contrast to the findings reported by Yang et al. [4] who observed little change in electrophoretic mobility as a function of pH for other food-grade TiO_2_ sources. Furthermore, the IEP value for E171 was 4.6. Yang et al. [4] found a variety of IEP values (i.e., from < 2.5 to 4.0) for five food-grade TiO_2_ sources, whereas Dudefoi et al. [27] reported a narrow range between 4.0 and 4.2 for food-grade TiO_2_ from other suppliers. These differences are probably due to the variable use of anionic phosphate-containing surfactants, as phosphate adsorbed to TiO_2_ surfaces might decrease the IEP [4, 27]. Our food-grade TiO_2_ was shown to be devoid of any surface coating with phosphate [5].

This is the first study to assess the interaction of food-grade TiO_2_ with mucus by coupling in vitro and in vivo approaches. For our in vitro experiments on HT29-MTX cells, the chosen concentration was of the same order of magnitude as that used in the recent study of Dorier et al. [6] who analyzed the interactions of E171 with Caco-2/HT29-MTX cells, in comparison with Caco-2 cells, during acute vs. repeated exposure. The authors reported that E171 accumulated more than P25 under both exposure scenarios, due to larger E171 agglomerates, which settled more quickly than those of the reference nanomaterial, leading to a higher level of cell exposure and cell response. They also concluded that E171 can cross the mucus layer, which was roughly imaged using Alcian Blue staining, since the two cellular models with and without mucus accumulated similar amounts of TiO_2_. Here, fluorescent staining with an anti-MUC5AC antibody and confocal microscopy enabled visualization of the mucus produced by HT29-MTX cells, as MUC5AC was shown to be the major gel-forming mucin in this in vitro model [31]. We showed that mucus did not form a uniform layer above the cell surface, but rather a patchy structure with a thickness of less than 5 µm. Even though mucus secreted by HT29-MTX cells has been described to form a dense gel layer, in some cases entirely covering the cell surface [40–42], our observations are in agreement with previous studies in which mucus patches were visualized instead [33, 43–45]. Here, we found that food-grade TiO_2_ particles formed sparsely distributed clusters throughout the sample undoubtedly embedded in these mucus “islands”. This confirms the in vitro barrier function of mucus against translocation of particles, mainly determined by their size, surface charge and surface hydrophobicity [21]. This is in contrast to the findings of Dorier et al. [6], but was already highlighted by Georgantzopoulou et al. [46] using a Caco-2/HT29-MTX co-culture and silver particles of similar size to the TiO_2_ used in this study (200 nm).

We compared the TiO_2_ mucus-related effects in rats after acute (1-week) or sub-chronic (60 days) oral daily administration of E171 vs. NM-105 at relevant exposure levels for humans (0.1 and 10 mg/kg bw/day). In vivo, mucus covers the intestinal epithelium but differently along the gastrointestinal tract. In particular, in rodents, mucus in the small intestine is single-layered and fills up the space between the villi and covers them, but is not attached to the epithelium and is penetrable to particles with the same size than that of bacteria [47]. In contrast, in the colon, a two-layered mucus system organization is generally described [48, 49]: the inner layer is densely packed, firmly attached to the epithelium, and devoid of bacteria, while the outer layer is movable, has an expanded volume and is heavily colonized by bacteria. However, Kamphuis et al. [50] recently proposed that the mucus layer in the distal colon of rodents covers the faeces instead of the epithelium. This faecal mucus layer confines the microbiota to the faeces and prevents it from remaining in empty distal colon. In the proximal colon, the mucus does not form a separating layer between bacteria and epithelium. These findings offer a new view of the mucus structure/function relationship. Besides its barrier properties, mucus is increasingly recognized to exert other key physiological functions essential for gut health. In particular, it provides a habitat for the gut microbiota since the wide diversity of mucin-derived carbohydrates can be utilized by bacteria as carbon sources [51–53] and/or preferential binding sites [54]. It was even suggested that such enormous repertoire could be at play in the region-specific colonization of bacteria in the gut [55]. Mucolytic bacteria, some of them being biomarkers in gut health and disease [56, 57], possess a large set of enzymes (glycosidases, sulphatases, sialidases…) to degrade mucin glycans and to harvest oligosaccharides for their own metabolism, thus conferring competitive advantage for mucosal surface colonization. Another important trait for adaptation of intestinal bacteria to the mucosal environment is their mucus-binding capacity, driven by specific determinants like pili and/or mucus-binding proteins in lactobacilli [58, 59].

Due to its trapping properties, mucus may act as a reservoir of TiO_2_ particles, leading to areas with a high local concentration. Owing to the mucus/microbiota interplay in the gut, the gut microbiota may be affected because of the reported in vitro bacterial toxicity of nano-sized TiO_2_ [60–62], and also food-grade E171 as recently shown by our group [63]. Indeed, there is growing evidence that the microbiota is a major player in food toxicology in the gut. Commensal bacteria have a capacity to metabolize xenobiotics and alter their toxicity to the host, as observed for drugs. Xenobiotics may themselves alter bacterial populations that colonize the gut, leading to dysbiosis and associated chronic diseases [64–66]. The consequences of oral exposure to TiO_2_ and other nanoparticles on the intestinal microbiota are largely unknown [21, 22, 67, 68]. Here, we focused on putative in vivo TiO_2_-induced changes to the fermentation products (SCFA) of commensal bacteria, because their effects, especially those of butyrate, on mucin synthesis and mucus characteristics have been described in vitro, ex vivo, and in vivo [19, 28, 69–71]. In particular, butyrate, propionate, and acetate, have been shown to increase *MUC2* mRNA synthesis in the LS174T cell line [28]. Butyrate increased *MUC2* mRNA levels at low concentrations (1 mM) but had no effect at moderate and high concentrations (5–15 mM). Stimulation with low concentrations of butyrate (1–2 mM) also increased MUC2 protein synthesis [28]. On the other hand, as seen above, mucus is an important substrate for the intestinal microbiota, especially SCFA-producing members [72–74] and considerable changes in mucus production would therefore result in altered SCFA profiles. In our study, exposure to food-grade TiO_2_ had little to no impact on the overall cecal composition of SCFA, including butyrate, irrespective of treatment duration (1-week vs. sub-chronic), suggesting that there were no substantial changes in mucus “quantity”. We then focused on mucus “quality” by assessing mucin *O*-glycosylation. Neither 1-week or sub-chronic exposure to food-grade TiO_2_ altered *O*-glycosylation of mucins from the small intestine and colon relative to untreated animals, first reinforcing the little impact on the “mucophilic” population of the gut microbiota. The only published study on the consequences of oral nanoparticle exposure to mucus deals with silver nanoparticles in rats [75]. Silver nanoparticle treatment resulted in a decrease in the level of neutral and acidic mucins in goblet cells, an increase in sialomucin levels, and a decrease in sulfomucin levels. We previously showed that changes in mucin *O*-glycan composition in rats may influence physico-chemical interactions and then condensation/bundling of mucin fibers, with a direct deleterious impact on the morphology and physical properties of the mucus network (i.e., loss in cohesive properties), and hence its protective barrier function [20]. In our study, the absence of mucin *O*-glycan alterations indicates that the protective function of mucus against particle uptake probably remained intact, even after sub-chronic oral exposure to food-grade TiO_2_.

## Conclusions

This study used a comprehensive approach to evaluate interactions between food-grade TiO_2_ (E171 additive) and intestinal mucus. Results were compared with those of the NM-105 (Aeroxyde P25) OECD reference nanomaterial. We showed region-specific trapping of food-grade TiO_2_ particles by mucus-secreting HT29-MTX intestinal epithelial cells, likely due to the patchy structure of mucus in this in vitro model. Cecal short-chain fatty acid profiles and mucin *O*-glycosylation patterns in the small intestine and colon were unchanged for E171 and also NM-105 after acute (1-week) and sub-chronic (60 days) oral exposure of rats at relevant exposure levels for humans (0.1 and 10 mg/kg body weight (bw)/day), suggesting the absence of a mucus barrier impairment under these “healthy gut” conditions. However, the protective functions of mucus are not static, as alterations in mucus production and composition have been shown to occur in response to microbial challenge, variations in diet, and intestinal disorders. These factors might influence the effects of the food additive E171. It should thus be informative to evaluate the risks of food-grade TiO_2_ uptake specifically in situations in which the intestinal barrier function is defective, in particular an impaired mucus barrier.

## Additional files

**Additional file 1.** Movie sequence showing the fluorescence distribution inside HT29-MTX structures from the substratum to the top before the addition of food-grade TiO_2_ (E171).

**Additional file 2.** Movie sequence showing the fluorescence distribution inside HT29-MTX structures from the substratum to the top 1 hour after the addition of food-grade TiO_2_ (E171).

## Abbreviations

BSA: bovine serum albumin
CPBM: Centre de Photonique Biomédicale
DLS: Dynamic Light Scattering
DMEM: Dulbecco’s modified Eagle’s minimal essential medium
EM: electrophoretic mobility
EU: European Union
EU JRC: European Union Joint Research Centre
FAO: Food and Agriculture Organization
FDA: Food and Drug Administration
FCS: fetal calf serum
HNO_3_: nitric acid
HyD: hybrid detectors
ICP-MS: Inductively Coupled Plasma Mass Spectrometry
IEP: isoelectric point
KOH: potassium hydroxide
MS: Mass Spectrometry
OECD: Organisation for Economic Cooperation and Development
PFA: paraformaldehyde
PBS: phosphate buffered saline
SCFA: short-chain fatty acids
TiO_2_: titanium dioxide
TiO_2_-NP: TiO_2_ nanoparticles
UV: ultraviolet
WHO: World Health Organization
